# Effects of load carriage on physiological determinants in adventure racers

**DOI:** 10.1371/journal.pone.0189516

**Published:** 2017-12-07

**Authors:** Alex de O. Fagundes, Elren P. Monteiro, Leandro T. Franzoni, Bruna S. Fraga, Patrícia D. Pantoja, Gabriela Fischer, Leonardo A. Peyré-Tartaruga

**Affiliations:** 1 Exercise Research Laboratory, Escola de Educação Física, Fisioterapia e Dança, Universidade Federal do Rio Grande do Sul, Porto Alegre, Rio Grande do Sul, Brazil; 2 Neurosciences and Rehabilitation Laboratory, Universidade Federal de Ciências da Saúde de Porto Alegre, Porto Alegre, Rio Grande do Sul, Brazil; 3 Centro de Desportos, Universidade Federal de Santa Catarina, Florianópolis, Brazil; 4 Post-Graduation in Pulmonology Science, Hospital de Clínicas de Porto Alegre, Universidade Federal do Rio Grande do Sul, Brazil; Universita degli Studi di Verona, ITALY

## Abstract

Adventure racing athletes need run carrying loads during the race. A better understanding of how different loads influence physiological determinants in adventure racers could provide useful insights to gauge training interventions to improve running performance. We compare the maximum oxygen uptake (VO2max), the cost of transport (***C***) and ventilatory thresholds of twelve adventure running athletes at three load conditions: unloaded, 7 and 15% of body mass. Twelve healthy men experienced athletes of Adventure Racing (age 31.3 ± 7.7 years, height 1.81 ± 0.05 m, body mass 75.5 ± 9.1 kg) carried out three maximal progressive (VO2max protocol) and three submaximal constant-load (running cost protocol) tests, defined in the following quasi-randomized conditions: unloaded, 7% and, 15% of body mass. The VO2max (unload: 59.7 ± 5.9; 7%: 61.7 ± 6.6 and 15%: 64.6 ± 5.4 ml kg^-1^ min^-1^) did not change among the conditions. While the 7% condition does neither modify the ***C*** nor the ventilatory thresholds, the 15% condition resulted in a higher ***C*** (5.2 ± 0.9 J kg^-1^ m^-1^; *P* = 0.001; *d* = 1.48) than the unloaded condition (4.0 ± 0.7 J kg^-1^ m^-1^). First ventilatory threshold was greater at 15% than control condition (+15.5%; *P* = 0.003; *d* = 1.44). Interestingly, the velocities on the severe-intensity domain (between second ventilatory threshold and VO2max) were reduced 1% equivalently to 1% increasing load (relative to body mass). The loading until 15% of body mass seems to affect partially the crucial metabolic and ventilatory parameters, specifically the ***C*** but not the VO2max. These findings are compatible with the concept that interventions that enhance running economy with loads may improve the running performance of adventure racing’s athletes.

## Introduction

The adventure racing (AR) consists of a multi-sports modality involving running, mountain-bike, canoeing, vertical techniques, and others. During the event, the athletes carry backpacks of different weights (5–10 kg), including obligatory equipment, in distances varying about 20–100 km. Despite the increasing popularity, few studies have analyzed the crucial aspects related to training workload and running performance with loads in these athletes [1].

The load carrying induces to a higher cost of transport (***C***) or energy cost, i.e., the energy spent per unit distance covered [2] in comparison to unload condition. However, when the ***C*** is scaled linearly to total mass (extra + body mass) no differences, and even reductions in energy expenditure with low loads are found [3, 4]. Possibly, the elastic mechanism is optimized for the loaded running [3]. However, metabolic data in maximal situations or at anaerobic threshold remain unknown.

The nutritional aspects, on the other hand, are extensively studied in AR due to its very demanding characteristic from the energetic point of view. A severe negative energetic balance in AR may produce adverse effects on immunological [5], renal [6], and muscular [7] systems. In fact, progressive intensive protein depletion has been verified during adventure races [7] accompanied by a negative energetic balance [8, 9]. Thus, the relevance of knowledge on metabolic requirements using loads has been discussed due to the evident impact on the energetic balance of these athletes [5–9].

The determination of intensities corresponding to certain metabolic domains is useful when planning and applying interval and continuous training methods. As for speed and distance, the load carriage would be a factor that affects the metabolic intensity domains. For example, when progressive load-carriage exercise is part of the training program, much larger training effects are evident than aerobic training alone [10, 11]. To the best of our knowledge, evidence-based recommendations for running training with loads are not established for AR participants.

Therefore, to date, little is still known about the ***C*** and ventilatory thresholds in the specific context of trained adventure athletes. We hypothesized that the differences in the submaximal intensities would be more noticeable with 15% load than 7%, due to energy-saving mechanism acting at low loads as previously shown [3]. Using a laboratory-based measures, we addressed two main research questions: i) is there an effect of load carriage on running maximum oxygen uptake (VO2max), ventilatory thresholds and ***C*** in AR athletes? ii) If loads affect these variables, is it possible to define predictive equations to estimate the crucial training intensity markers based on extra load?

## Materials and methods

### Participants

Based on a minimum increase in ***C*** of 5% (~0.3 J kg^-1^ m^-1^), a coefficient of variation of 5%, an alpha error of 0.05 and a power of 90%, the minimal number of athletes required for the group was 11. Twelve healthy men, who were national level athletes of AR (age 31.3 ± 7.7 years, height 1.81 ± 0.05 m, body mass 75.5 ± 9.1 kg, training volume 39.12 ± 9.02 km per week, AR experience 64 ± 49 months), carried out three maximal progressive (VO2max protocol) and three submaximal constant-load (running cost protocol) tests, defined in the following quasi-randomized conditions: unloaded, 7% and, 15% of body mass. We choose percent loads due to inherent effects of absolute load on performance. Also, we settle these percent loads because are the loads usually used in AR [11]. All participants gave their written informed consent to participate in the study. All procedures followed were in accordance with the ethical standards and with the Helsinki Declaration of 1975, as revised in 2008, and were approved by the responsible local Ethics Committee of the Universidade Federal do Rio Grande do Sul on human experimentation.

### Experimental design

**Fig 1:**
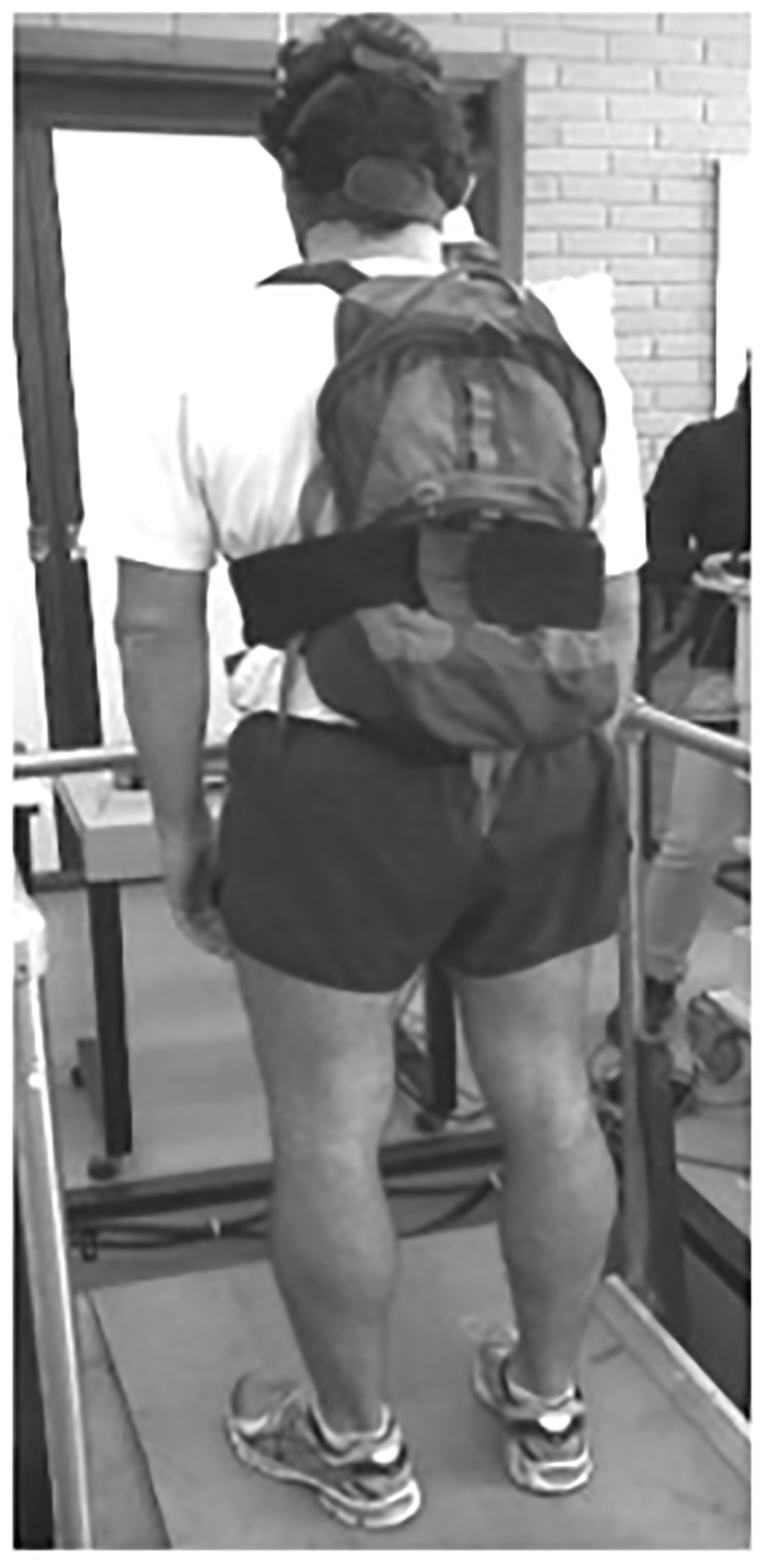
The athlete and his backpack with the extra load.

During the preliminary visit, athletes were familiarized with all loads, equipment, and protocols. All tests were separated by about 2–4 days. Firstly, the three maximal running tests at 0, 7% and 15% of individuals’ body mass were randomized in three visits and, in the fourth visit, the submaximal tests were again randomized. The athletes used their backpacks to perform the bouts with the extra load. The backpack position was set between the first thoracic and lumbar vertebra, and it was fixed to avoid excessive oscillation (Fig 1). In all tests, the heart rate (Polar, Kempele, Finland), end-tidal partial pressure of oxygen, end-tidal partial pressure of carbon dioxide, oxygen uptake, carbon dioxide output and ventilation per minute (MEDGRAPHICS, CPX/D, Diagnostic Systems, Saint Paul, Minnesota, USA) were measured continuously. The gas data were registered breath-by-breath. Temperature, atmospheric pressure and humidity in the laboratory were 20 ± 2°C, 1026 ± 10 mmHg, and 50 ± 8%, respectively. 6–20 Borg’s ratings of perceived exertion scale (RPE) was shown to the athletes during the last 30 s of each stage (maximal tests) and just after the end of submaximal tests. Each athlete received detailed instructions about the use of the scale before the beginning of the first test. The total time at each maximal test was 30 minutes, and 1 hour to the submaximal protocol.

#### Maximal test

Before the trials, the athletes performed a warm-up walking on a treadmill (QUINTON, ST55, New York, USA) inclined at 1% for five minutes at 6.0 km h^-1^ [12]. During the warm-up before the tests with load, the backpack with the respective load (7 or 15% of body mass) was adjusted. They were familiarized to treadmill exercise. The initial speed of the maximal tests was 6.0 km h^-1^, and the increment was 1.0 km h^-1^ per minute until subjects reached volitional exhaustion.

#### Submaximal tests

Initially, resting oxygen uptake was measured in orthostasis, during 5 min. The individuals were asked to carry-out a warm-up for five minutes walking on a treadmill. Taking into account the individual outcomes from maximal tests, the athletes carried out the submaximal tests at the intensity associated with 10% below the second ventilatory threshold according to the respective condition (0%, 7%, and 15% of the individual’s body mass), all on the same day. The duration of each test was 6 min. On average, 10 minutes of rest between submaximal tests were enough to achieve the initial heart rate and oxygen consumption. The rates of oxygen consumption and carbon dioxide production were measured continuously during each trial. The heart rate in all submaximal tests was not greater than 80 percent of the maximal heart rate. Besides, the respiratory exchange ratio was also monitored achieving values lower than one.

### Data analysis

#### Maximal tests

The VO2max, the velocity associated with VO2max (vVO2max), maximal RER, maximal heart rate, first and second ventilatory thresholds, and velocity associated with first and second ventilatory thresholds (v1Tvent and v2Tvent, respectively) were determined using computerized indirect calorimetry system [13].

The highest average of five oxygen consumption values was interpreted as the VO2max [14]. The value was considered valid when, at least, one of following criteria was observed: i) estimated maximal heart rate; ii) plateau on oxygen consumption with concomitant increase in the speed (all subjects attained the true VO2max); iii) respiratory exchange ratio greater than 1.1; iv) rating of perceived exertion greater than 17 (very hard) relative to Borg scale.

The first and second ventilatory thresholds were determined according to the method proposed by [15]. The first ventilatory threshold (also denominated as individual ventilatory threshold) was determined from the first increase in ventilation-minute with a rapid rise in the ventilatory equivalent of oxygen consumption with no concomitant increase in the ventilatory equivalent of carbon dioxide production curve. The second ventilatory threshold (also denominated as respiratory compensation point) was defined as follows: i) a systematic increase in the ventilatory equivalent of oxygen consumption; ii) a concomitant nonlinear increase in the ventilatory equivalent of carbon dioxide production; and iii) a reduction in the difference in the inspired and end-tidal oxygen pressure. The ventilatory thresholds were determined in a blinded way by two independent evaluators.

#### Submaximal tests

The submaximal oxygen uptake and heart rate were averaged from the last 60 s of the test [16]. The running economy was denoted by ***C***, expressed in J kg^-1^ m^-1^. For that, we divided the net metabolic rate (gross—stand metabolic rate) by speed and we converted oxygen in ml to Joules relative to combustion enthalpy of substrates resulting from oxidation observed indirectly from respiratory exchange ratio [17]. The metabolic rate (ECO) was also calculated and expressed in ml kg^-1^ min^-1^.

The maximal and submaximal metabolic power values were normalized to body mass and expressed in ml kg^-1^ min^-1^. Recently, we showed that the relationship between metabolic parameters and performance is independent of how the parameters are relativized in runners [18–20]. All data can be seen in the supplementary material (S1 Table).

### Statistics

The Shapiro-Wilk test was used to verify data normality. We performed the descriptive statistics calculating mean ± standard deviation. The Pearson product-moment correlation test was carried out in order to test the relationship among the physiological determinants of performance (VO2max, ***C*** and, ventilatory threshold) with different load conditions. The linear regression analysis was used to estimate the speeds associated empirically with ventilatory thresholds when carrying loads. Possible differences between conditions (0, 7 and 15% of body mass) were analyzed using the repeated-measures analysis of variance (ANOVA) with Bonferroni post hoc test. To verify the possibility of violation of the assumption of sphericity, we applied the Mauchly test using the Greenhouse-Geisser correction for all analyses. Significance was accepted at *P* ≤ 0.05, statistical power was 90%, and the analyses were performed in Statistical Package for Social Sciences version 20.0 (SPSS, Chicago, Illinois, USA). We used the Cohen’s *d* coefficient to determine the effect sizes [21]. We determined the differences in proportions using the rule of thumb criteria set out by Hopkins: trivial (< 0.2), small (0.2–0.6), moderate (0.6–1.2), or large (> 1.2).

## Results

**Fig 2:**
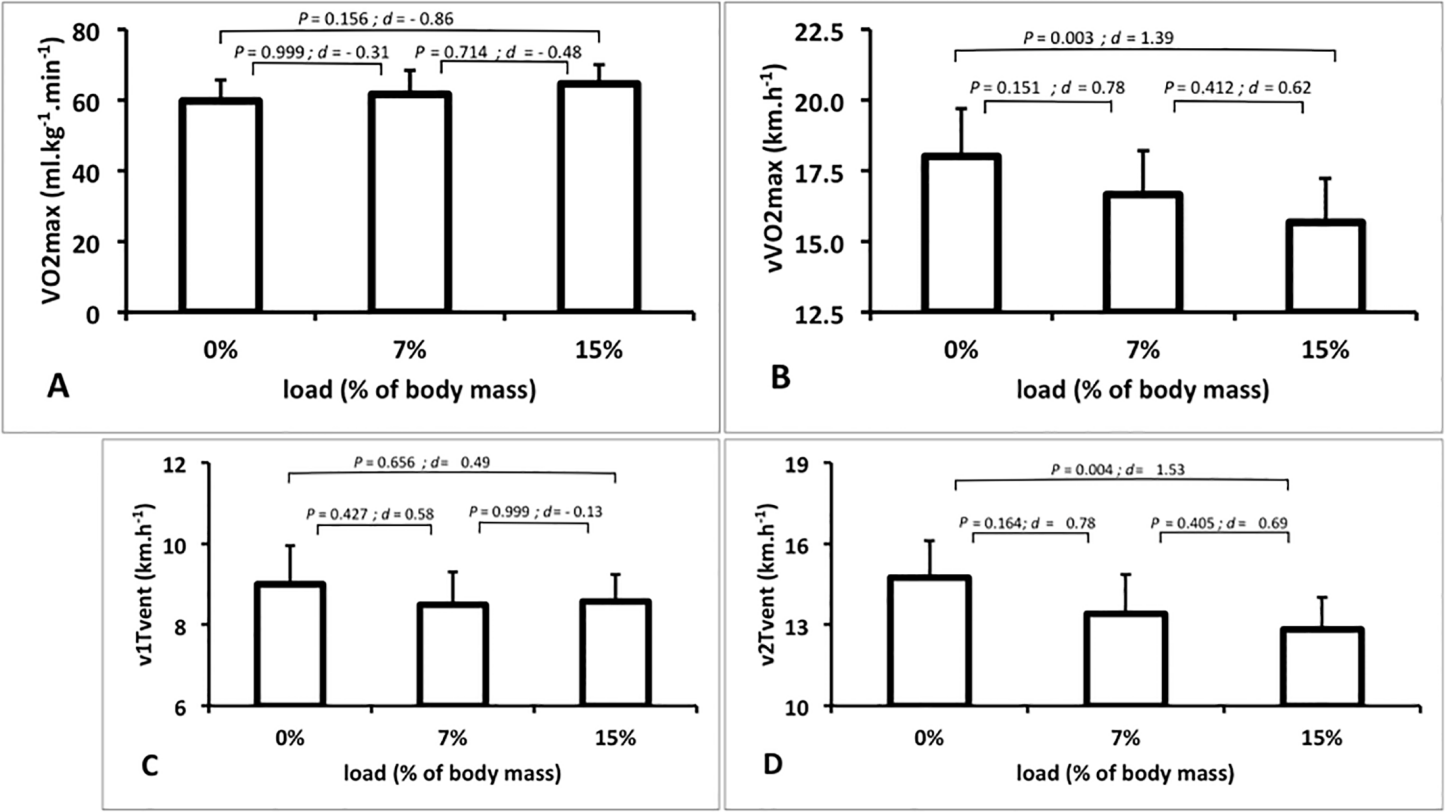
Mean and standard deviation of VO2max (A), velocities associated with VO2max (vVO2max, B), first (v1Tvent, C) and second (v2Tvent, D) ventilatory thresholds at different conditions (0%: unloaded; 7% of body mass; and 15% of body mass). *P*’s and effect sizes (Cohen’s *d*) are also presented.

The physiological data are presented in Table 1. The average for 7 and 15% loads carried in the maximal and submaximal tests were 5.29, *s* = 0.64 kg and 11.33, *s* = 1.37 kg, respectively. ANOVA showed a general effect of load on vVO2max (*P* = 0.005, Fig 2B), which decreased 13% between 0 and 15% load. Despite no significant statistical differences among VO2max values (Fig 2A), a large effect size (0.86) was observed between 0 and 15% load.

**Table 1:** Mean, standard deviation, ANOVA, post hoc (Bonferroni) and Cohen’s *d* effect size results from maximal (VO2max protocol) and submaximal (running cost protocol) tests. Numbers in bold represent *P* < 0.05.

	0%	7%	15%	ANOVA		Bonferroni (effect size)		
				F	*P*	*P* 0–7%	*P* 0–15%	*P* 7–15%
Load (kg)	--	5.3 ± 0.6	11.3 ± 1.4					
*VO2max protocol data*								
VO2max (ml kg^-1^ min^-1^)	59.7 ± 5.9	61.7 ± 6.6	64.6 ± 5.4	2.05	0.144	0.999 (0.31)	0.156 (0.86)	0.714 (0.48)
HRmax (bpm)	183 ± 9	181 ± 8	181 ± 12	0.15	0.863	0.999 (0.23)	0.999 (0.18)	0.999 (0.00)
vVO2max (km h^-1^)	18.0 ± 1.7	16.7 ± 1.6	15.7 ± 1.6	6.36	**0.005**	0.151 (0.78)	**0.003** (1.39)	0.412 (0.62)
RERmax	1.14 ± 0.07	1.13 ± 0.09	1.15 ± 0.10	0.24	0.790	0.999 (0.12)	0.999 (0.11)	0.999 (0.21)
1Tvent (ml kg^-1^ min^-1^)	33.2 ± 3.8	37.5 ± 4.1	39.3 ± 4.6	6.75	**0.003**	**0.050** (1.08)	**0.003** (1.44)	0.904 (0.41)
2Tvent (ml kg^-1^ min^-1^)	51.8 ± 4.3	55.5 ± 6.3	56.5 ± 6.9	2.07	0.140	0.425 (0.68)	0.184 (0.81)	0.999 (0.15)
v1Tvent (km h^-1^)	9.0 ± 0.9	8.5 ± 0.8	8.6 ± 0.7	1.30	0.287	0.427 (0.58)	0.656 (0.49)	0.999 (0.13)
v2Tvent (km h^-1^)	14.8 ± 1.4	13.7 ± 1.4	12.8 ± 1.2	6.24	**0.005**	0.164 (0.78)	**0.004** (1.53)	0.405 (0.69)
1Tvent% (%)	55.8 ± 5.9	61.1 ± 6.5	60.8 ± 5.9	0.68	0.514	0.861 (0.85)	0.999 (0.84)	0.999 (0.04)
2Tvent% (%)	87.1 ± 5.3	90.0 ± 5.3	87.5 ± 8.3	2.85	0.072	0.127 (0.54)	0.156 (0.05)	0.999 (0.35)
v1Tvent% (%)	50.2 ± 5.4	51.3 ± 6.1	55.0 ± 4.3	2.72	0.081	0.999 (0.19)	0.098 (0.98)	0.290 (0.70)
v2Tvent% (%)	82.2 ± 6.4	82.4 ± 9.1	82.3 ± 7.8	0.01	0.998	0.999 (0.02)	0.999 (0.01)	0.999 (0.01)
HR at 1Tvent (bpm)	127 ± 12	130 ± 13	132 ± 15	0.54	0.585	0.999 (0.23)	0.930 (0.36)	0.999 (0.14)
HR at 2Tvent (bpm)	167 ± 13	167 ± 10	166 ± 12	0.01	0.989	0.999 (0.00)	0.999 (0.07)	0.999 (0.09)
RPE at 1Tvent	9.2 ± 1.2	9.1 ± 0.9	9.9 ± 1.6	1.62	0.213	0.999 (0.09)	0.453 (0.49)	0.335 (0.61)
RPE at 2Tvent	14.7 ± 2.1	13.9 ± 2.5	14.4 ± 2.6	0.36	0.701	0.999 (0.34)	0.999 (0.12)	0.999 (0.19)
RPE at VO2max	18.7 ±1.5	18.2 ± 1.3	18.4 ± 1.4	0.51	0.607	0.968 (0.35)	0.999 (0.20)	0.999 (0.14)
*Running cost protocol data*								
ECO (ml kg^-1^ min^-1^)	42.1 ± 6.0	45.3 ± 6.7	48.1 ± 9.7	1.87	0.170	0.941 (0.50)	0.186 (0.74)	0.999 (0.33)
*C* (J kg^-1^ m^-1^)	4.0 ± 0.7	4.6 ± 0.7	5.2 ± 0.9	7.97	**0.001**	0.115 (0.85)	**0.001** (1.48)	0.229 (0.74)
Speed (km h^-1^)	13.3 ± 1.2	12.3 ± 1.3	11.5 ± 1.1	1.98	0.154	0.185 (0.79)	0.530 (1.56)	0.999 (0.66)
ECO% (%)	76 ± 8	80 ± 12	78 ± 11	0.32	0.725	0.999 (0.39)	0.999 (0.20)	0.99 (0.17)
HR_ECO (bpm)	162 ± 15	161 ± 16	158 ± 15	0.17	0.846	0.999 (0.06)	0.999 (0.26)	0.999 (0.19)
RPE_ECO	11.0 ± 2.0	11.6 ± 2.0	11.8 ± 1.9	0.66	0.525	0.999 (0.29)	0.842 (0.41)	0.999 (0.10)

Note: VO2max: maximal oxygen consumption; HRmax: maximal heart rate; RERmax: maximal respiratory exchange ratio; vVO2max: velocity at VO2max; 1Tvent: first ventilatory threshold; 2Tvent: second ventilatory threshold; v1Tvent: velocity at first ventilatory threshold; v2Tvent: velocity at second ventilatory threshold; 1Tvent%: percent first ventilatory threshold; 2Tvent%: percent second ventilatory threshold; v1Tvent%: percent velocity associated with first ventilatory threshold; v2Tvent%: percent velocity associated with second ventilatory threshold; HR at 1Tvent: heart rate at first ventilatory threshold; HR at 2Tvent: heart rate at second ventilatory threshold; RPE at 1Tvent: rating of perceived exertion at first ventilatory threshold; RPE at 2Tvent: rating of perceived exertion at second ventilatory threshold; and RPE at VO2max: rating of perceived exertion at maximal oxygen consumption. ECO: metabolic rate; *C*: cost of transport; ECO%: percent metabolic rate; HR_ECO: heart rate during submaximal test; RPE_ECO: rating of perceived exertion during submaximal test.

**Fig 3:**
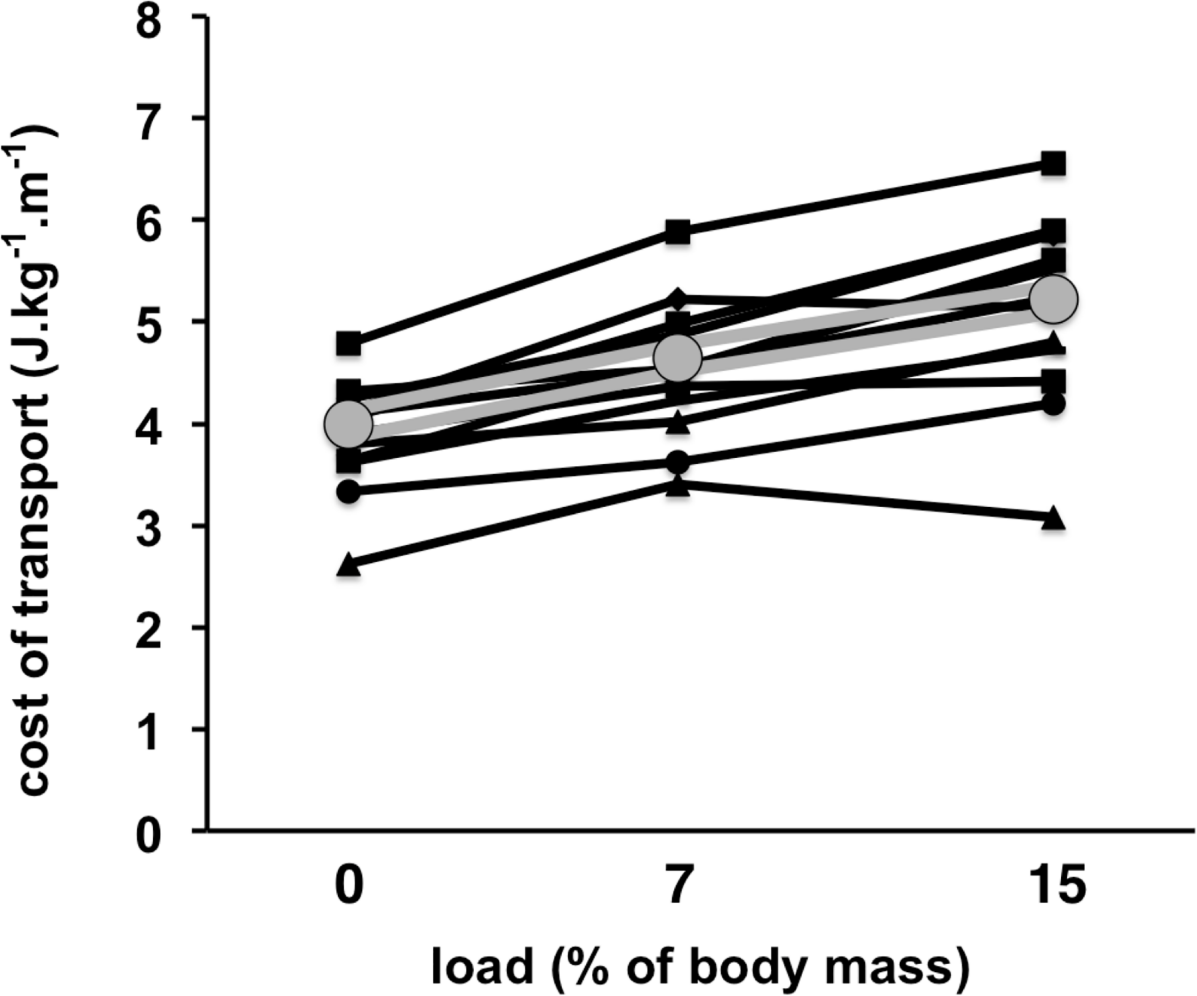
Cost of transport per athlete (black lines) and the average value (gray double line) at unloaded condition (0), carrying loads of 7 and 15% of body mass.

First ventilatory threshold values significantly increased (*P* = 0.003) while the velocity associated, v1Tvent, did not differ among loads (*P* = 0.287, Fig 2C). On the contrary, second ventilatory threshold values were similar (*P* = 0.140) while the v2Tvent decreased 13.5% (*P* = 0.005, Fig 2D), as observed for the vVO2max. Heart rate, rating of perceived exertion at first and second ventilatory threshold, and VO2max did not differ (*P* > 0.05) among load conditions (Table 1). In contrast, ***C*** resulted to be significantly greater (+30%; *P* = 0.001; *d* = 1.48) at 15% load and greater (+13%; *P* = 0.115; *d* = 0.85) at 7% load compared to the unloaded running submaximal test (Fig 3).

**Fig 4:**
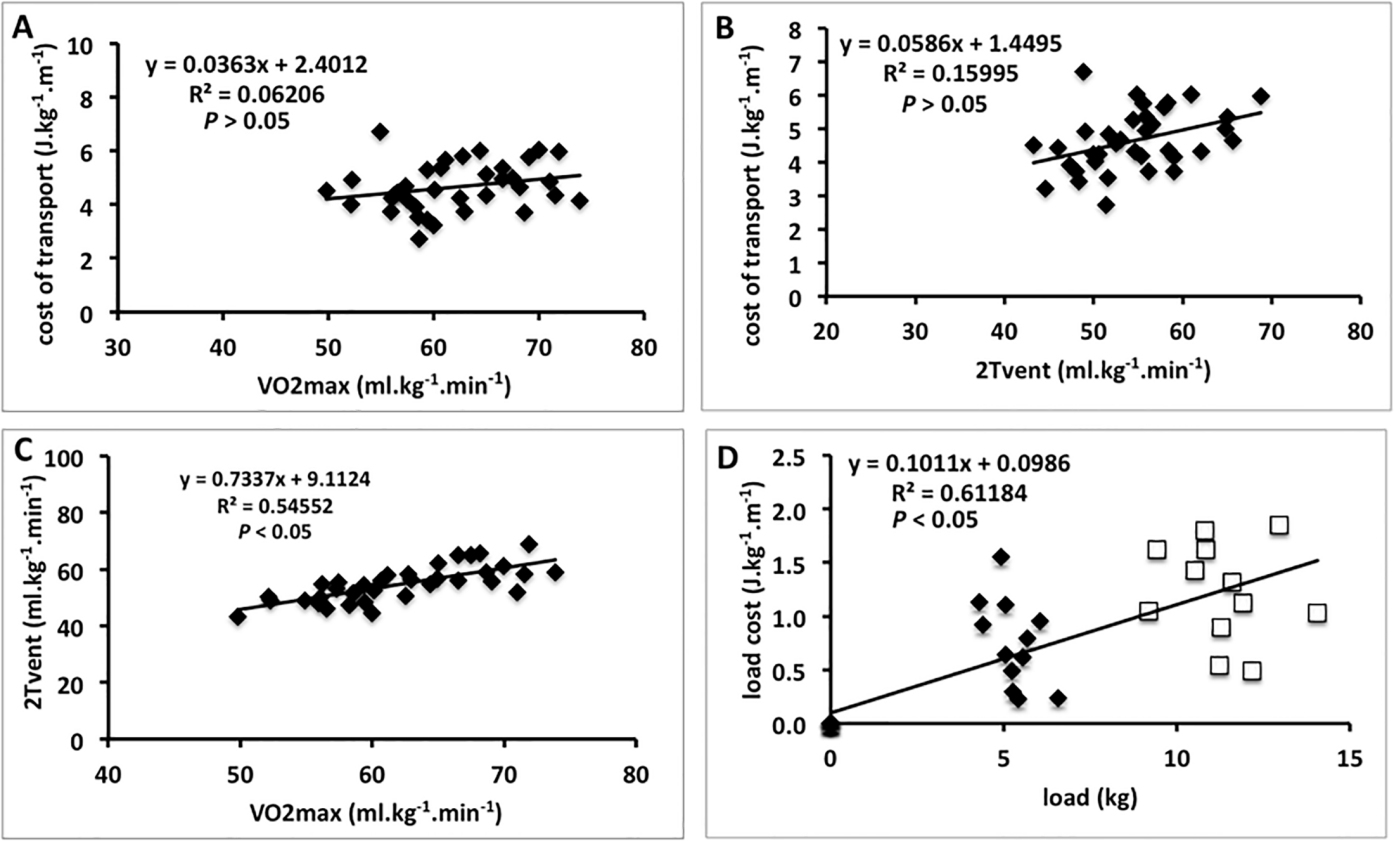
Scatterplot between physiological variables (*n* = 12 subjects). A) The cost of transport in function of maximal oxygen consumption (VO2max) and, B) in function of second ventilatory threshold (2Tvent). C) The 2Tvent in function of VO2max. D) The metabolic cost of load (load cost = loaded−unloaded) in function of absolute load in kg.

A significant moderate correlation was observed between the second ventilatory threshold and VO2max (*r* = 0.74, *P* <0.05, Fig 4C). Interestingly, the extra-cost (the metabolic cost to transport the backpack) seems to be a function of backpack weight (*r* = 0.78, *P* <0.05, Fig 4D).

We estimated the relative intensity of training (reduction on v1Tvent, v2Tvent, and vVO2max due to load carriage) by the linear regression model. The linear regression between reduction of the v1Tvent and load was weak and non significant (*r*^*2*^ = 0.05; *P* >0.05). On the other hand, the estimation of the reduction of the vVO2max (in km h^-1^) and v2Tvent in function of load carriage were as follows: vVO2max (load, kg) = -0.1932 *x* load– 0.1523 (*r*^*2*^ = 0.54; *P* < 0.05; SEE = 0.2186 km h^-1^); v2Tvent (load, kg) = -0.1676 *x* load– 0.1549 (*r*^*2*^ = 0.68; *P* <0.05, SEE = 0.1422 km h^-1^).

## Discussion

Our first and most important research question was to determine whether there is an effect of load carriage on running VO2max, ventilatory thresholds, and ***C*** in experienced athletes of AR. We found that VO2max and second ventilatory threshold were affected similarly, that is, values reached were similar while the velocities associated were significantly reduced. We also accepted our hypothesis that adventure racers have higher differences in the ***C*** when carrying loads of 15% than 7% of body mass in comparison to the unloaded condition. Our second research question was related to testing predictive equations to estimate specific training intensity for adventure racers from the loads used. To the best of our knowledge, this is the first study investigating the effects of load carriage on running VO2max, ***C*** and ventilatory threshold in adventure racers.

### About the method

What remains to be established is how much of the reduction in velocity is related to load carriage and whether a similar reduction also carries out with different intensities. These are important issues given that loaded running may play a role in a substantial proportion of metabolic requirements during the AR. To address these questions, we chose to focus on backpack weights of 7 and 15 percent of body mass as these loads are frequent in adventure races [18]. As the individual speed is critical to organizing the strategy of AR teams [22], we chose to estimate the velocities associated with different performance threshold.

### Physiological meaning of findings

The adventure racers’ v1Tvent did not change carrying loads until 15% of body mass, but the metabolic rate expended at that level was increased with loading condition. One possible explanation refers to the reduced running economy (***C***) at similar intensity found out in our study. Also, on the other hand, maintain the v1Tvent even with a higher oxygen consumption may be related to the intensity frequently used for these athletes in their races (120–130 bpm [8]), close to the heart rate at first ventilatory threshold found here (127–132 bpm at first ventilatory threshold). The maximal heart rate, respiratory exchange ratio and rating of perceived exertion were not modified in the different conditions due to the substantial reduction in the vVO2max, which is in line with previous studies [23, 24].

At higher intensities, the vVO2max and v2Tvent were reduced as expected. We speculated that performance at the severe intensity domain was not worsened due to an optimization in the elastic bouncing of running. Since the total load is a crucial factor to explain the higher efficiency in larger animals due to minor hysteresis loss [25], we suggest that when using backpacks, the adventure racers have the muscle-tendon unities more loaded showing also the landing-takeoff asymmetry more elastic as recently proposed [26]. Direct evidence of animal studies [27] and indirect evidence in humans [3] support this hypothesis. Nevertheless, although we have measured the cost of carrying loads during locomotion, the function of the muscle-tendon unit cannot be ascertained in this study. Thus, the added mechanical work due to elastic bouncing with loads remains unknown, and the potential impact of loading at the level of muscle-tendon units requires further research.

In our study, ***C*** increased with the extra load. However, the effects of load on the ***C*** in the scientific literature are inconclusive [3, 4, 18]. There are methodological differences in the studies mentioned above that might partly explain different findings. One important issue refers to the way of expressing the energy expenditure, especially concerning the mass normalization. In the studies where the economy increased with load (e.g., Abe et al. [3]), the metabolic value was normalized to total mass. This procedure does not permit assessing the economy mainly related to muscles involved in the movement [2].

The ventilatory thresholds are considered good predictors of long-distance running performance [15]. In the current study, the athletes obtained moderate to high velocities [28] associated with the second ventilatory threshold (0% = 14.8, *s* = 1.4 km h^-1^; 7% = 13.7, *s* = 1.4 km h^-1^; 15% = 12.8, *s* = 1.2 km h^-1^). The second ventilatory threshold expressed as a percentage of VO2max were in the range of 87–90% VO2max. These results demonstrate that the athletes are aerobically well trained. Again, we explore the effects of velocities associated with the ventilatory threshold, and these determinations may be useful to plan training programs in order not just to maximize physiological adaptations but also to reduce the probability of susceptibility to overreaching and overtraining in this sport [29, 30]. From a practical point-of-view, this study provides an interesting outcome related to the one-to-one percent ratio between running velocity and extra-load (as a percentage of body mass) in the severe domain. In other words, the athlete of AR needs to pay attention that to each percent of the increase in the backpack’s load, the speed needs to be reduced at the same percentage, to maintain the same metabolic rate. These findings suggest that interventions that enhance the running economy (for example, strength training) may increase the athletes’ performance of AR.

### Predictive equations

Specifically, our predictive equations offer a valuable tool to control the training intensity when the load is manipulated. The average reductions of the velocities associated with VO2max due to increasing load were 8 and 15% (for 7 and 15% load, respectively), and of the velocities associated with second ventilatory threshold were 10 and 15%. Interestingly, these average values indicate a constant ratio equal to one between the percent velocity reduction (due to loading) and the percent load increase. Stated in other terms, 1% of load increase (relative to body mass) is equivalent to 1% of velocity reduction (relative to running velocity without extra load). This relationship seems to be constant only on severe intensity domain, between the second ventilatory threshold and VO2max.

Although it produces only empirical predictive equations, this approach is biologically meaningful and provides a useful framework for planning and developing specific training models to adventure racers. Furthermore, many attempts of estimating the ventilatory threshold parameters were not successful in the literature, showing an underestimation in a broad sample of endurance athletes (141 subjects, but taking part 8 adventure racers only). And, when validated, these equations are neither specific to AR [31] nor taking into account the loaded conditions [28, 32].

The experimental protocol we undertook has limitations that must be discussed. The load position is a crucial variable of ***C***, and we used only loads on the shoulders. Although loading subjects on their shoulders had a greater negative impact on ***C*** than placing the extra load around their waists [18], backpacks are the typical way of carrying the load in AR. One important limitation is about the time-dependent effects of load on physiological parameters studied here. The adventure races are performed during 4–5 days. Therefore, the mitochondrial function is deteriorated during the race [5], probably intensifying the negative effects of load on ***C***.

Moreover, the heart rate is qualitatively reduced in the second half of races [8]. These differences show that our results are limited to regular training and race’ starting phases. Future work can use this original study as an important starting point in the quest to improve our understanding of the physiological adaptations to specific training by using loads and their repercussions on race performance. We also suggest future studies analyzing running’s biomechanical alterations (stride length and frequency) under different loads in AR athletes.

The results of the present study showed collectively the preservation of running primary physiological parameters of adventure racers using loads, specifically, demonstrating the maintenance of VO2max, ***C*** (at 7% of body mass) and second ventilatory threshold, and the increase of the first ventilatory threshold (interestingly without differences in the v1Tvent). The metabolic cost of transporting 1 kilogram of body mass per meter of running (***C***) was impaired with 15% load only.

## Conclusion

The most striking findings of this cross-sectional study are as follows: (i) The VO2max and second ventilatory threshold remain unchanged, and the responses of first ventilatory threshold and ***C*** were greater at 15% of body mass in comparison to unloaded condition; (ii) at severe metabolic domain (from second ventilatory threshold to VO2max), the iso-metabolic speeds were reduced 1% equivalently to 1% increasing load (relative to body mass); (iii) the ***C*** of carrying 7% and 15% (of body mass) loads for AR athletes are 4.6 J kg^-1^ m^-1^ (1.10 cal kg^-1^ m^-1^) and 5.2 J kg^-1^ m^-1^ (1.24 cal kg^-1^ m^-1^), respectively; and for the unloaded condition, the ***C*** is 4.0 J kg^-1^ m^-1^ (0.96 cal kg^-1^ m^-1^).

Moreover, the regression model presented here is convenient for field use by adventure racers, as it requires only the information of the load carried on backpacks. These findings could be further used to optimize the performance of these athletes by individualizing training intensities related to load carriage.

## Supporting information

S1 Table General dataset. (XLSX)
